# Identification of Chilling-Responsive Genes in *Litchi chinensis* by Transcriptomic Analysis Underlying Phytohormones and Antioxidant Systems

**DOI:** 10.3390/ijms23158424

**Published:** 2022-07-29

**Authors:** Xiaoting Zhang, Hao Liu, Lijie Huang, Biyan Zhou

**Affiliations:** Key Laboratory of Biology and Genetic Improvement of Horticultural Crops (South China), Ministry of Agriculture and Rural Affairs, College of Horticulture, South China Agricultural University, Guangzhou 510642, China; huanxiw@126.com (X.Z.); huanglj93@sina.cn (H.L.); maomihaoge@163.com (L.H.)

**Keywords:** chilling, litchi, antioxidant system, transcriptome, hormone, PLS-SEM

## Abstract

Litchi (*Litchi chinensis* Sonn.) is an important subtropical and tropical evergreen fruit tree that is seriously affected by chilling stress. In order to identify genes that may be involved in the response to chilling in litchi, we investigate the physiological and biochemical changes under chilling stress and construct 12 RNA-Seq libraries of leaf samples at 0, 4, 8, and 12 days of chilling. The results show that antioxidant enzymes are activated by chilling treatments. Comparing the transcriptome data of the four time points, we screen 2496 chilling-responsive genes (CRGs), from which we identify 63 genes related to the antioxidant system (AO-CRGs) and 54 ABA, 40 IAA, 37 CTK, 27 ETH, 21 BR, 13 GA, 35 JA, 29 SA, and 4 SL signal transduction-related genes. Expression pattern analysis shows that the expression trends of the 28 candidate genes detected by qRT-PCR are similar to those detected by RNA-Seq, indicating the reliability of our RNA-Seq data. Partial Least Squares Structural Equation Modeling (PLS-SEM) analysis of the RNA-Seq data suggests a model for the litchi plants in response to chilling stress that alters the expression of the plant hormone signaling-related genes, the transcription factor-encoding genes *LcICE1*, *LcCBFs*, and *LcbZIPs*, and the antioxidant system-related genes. This study provides candidate genes for the future breeding of litchi cultivars with high chilling resistance, and elucidates possible pathways for litchi in response to chilling using transcriptomic data.

## 1. Introduction

Chilling is one of the main abiotic stresses affecting the growth and development of plants, geographical distribution, and production [1,2]. It can affect plant growth and development by changing the membrane system, cell osmotic conditions, metabolism, and antioxidant systems [3]. At present, chilling injury is a serious natural disaster in agriculture [4] that leads to a significant reduction in the yield of field crops [5]. Therefore, it is particularly important to investigate the mechanism of chilling injury and the chilling adaptation strategies of plants. These chilling adaptation strategies include chilling resistance underlying physiological and biochemical changes [6], a complex gene network of chilling responses, and post-transcriptional modifications [7,8]. At present, genes functioning in relation to chilling resistance have been identified [9]. Chilling resistance in arabidopsis has been improved by means of genetic engineering [10].

Litchi (*Litchi chinensis* Sonn.) is native to China and is a fruit tree of the Sapinaceae family [11]. It is an important subtropical and tropical evergreen fruit tree in Southeast Asia. However, chilling injury is one of the main factors that restricts litchi production. Hence, it is important to investigate the mechanism of litchi in response to chilling so as to find ways to increase its resistance. RNA sequencing (RNA-Seq) technology is a powerful tool for transcriptomic studies. This technique has been used to elucidate puffing disorder in citrus [12], to investigate transcriptomic changes in winter wheat [13], to elucidate transcriptomic responses of Lanzhou lily to drought stress [14], and to identify genes involved in low-temperature-induced senescence, as well as ROS-induced flowering in litchi [15,16]. However, up till now, no RNA-Seq data on litchi plants in response to chilling have been published.

In this study, we use RNA-Seq technology to identify differentially expressed genes (DEGs) which might be potentially involved in the chilling resistance of litchi’s underlying phytohormones and antioxidant systems. We assume that hormone-related DEGs might be directly or indirectly related to antioxidant system-related DEGs to regulate litchi’s response to low temperature. Hence, we perform Partial Least Squares Structural Equation Modeling (PLS-SEM) analysis to quantify the relationship among the biological processes based on the transcript levels of the DEGs [17]. The aim of this work is to elucidate pathways for litchi to adapt to chilling stress by phytohormones and antioxidant system-related genes.

## 2. Results

### 2.1. Phenotype of Litchi Leaves under Chilling Stress

**Figure 1:**
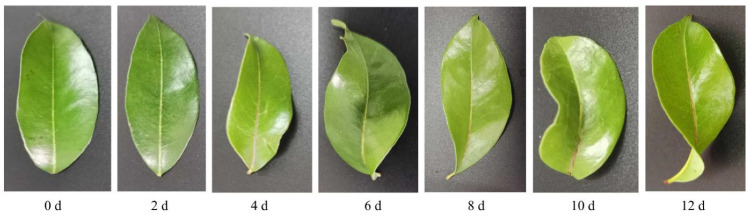
Leaf phenotype of ‘Huaizhi’ litchi trees under chilling stress. ‘Huaizhi’ litchi trees were transferred to a growth chamber. Temperature of the chambers dropped from 22 °C/17 °C (day/night temperature, 12 h day and 12 h night) to 2 °C in 4 days, and then remained at 2 °C for another 8 days. The days represent the samples of ‘Huaizhi’ litchi leaves on 0 d, 2 d, 4 d, 6 d, 8 d, 10 d, and 12 d under low-temperature conditions.

Figure 1 shows the morphological changes in litchi leaves under chilling stress. It was found that litchi leaves began to curl and the veins turned red from 4 d of treatment.

### 2.2. Physiological and Biochemical Changes in Response to Chilling Stress in Litchi Leaves

**Figure 2:**
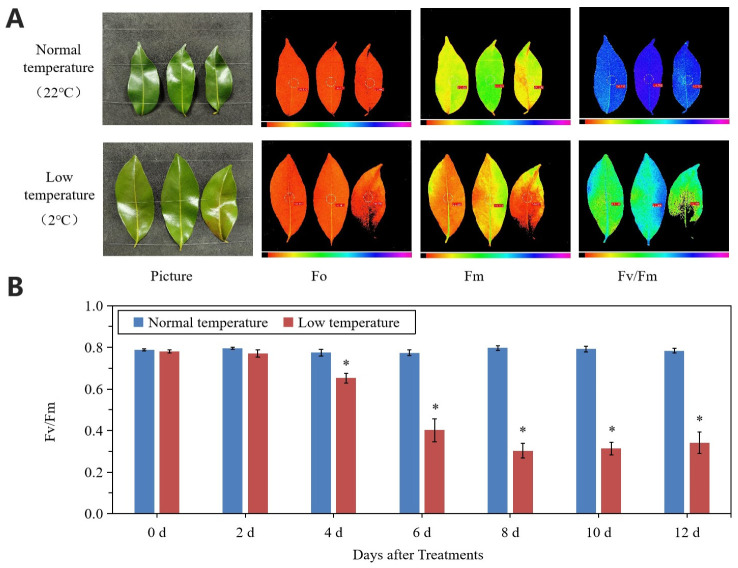
Changes in leaf chlorophyll fluorescence parameters under normal- and low-temperature conditions. (**A**) Phenotype and chlorophyll fluorescence imaging for parameters Fo, Fm, and Fv/Fm for leaves of ‘Huaizhi’ at normal temperature as control and low temperature as treatment at the time point of day 12. (**B**) Fv/Fm values of litchi leaves under low-temperature and normal-temperature conditions. Asterisks (*) indicate significant difference between treatment and control at the same time point (*p* ≤ 0.05, *n* = 3, Student’s *t*-test).

Chilling stress reduced the Fv/Fm values of litchi leaves (Figure 2A,B). From day 2, Fv/Fm values decreased and at day 6 to 12, the values were around 0.30, while those of the values at normal temperature remained at a high level above 0.75.

**Figure 3:**
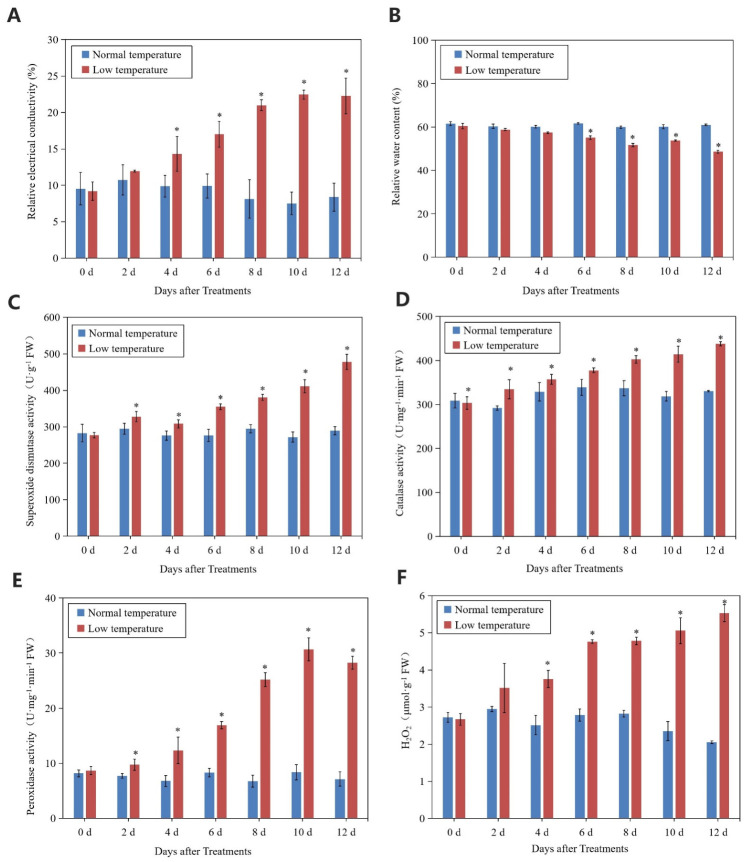
Changes in relative conductivity (**A**), relative water content (**B**), SOD activity (**C**), CAT activity (**D**), POD activity (**E**), and H_2_O_2_ content (**F**) of leaves in ‘Huaizhi’ litchi trees under normal- and low-temperature conditions. ‘Huaizhi’ litchi trees were transferred to a growth chamber. Temperature of the chamber dropped from 22 °C/17 °C (day/night temperature, 12 h day and 12 h night) to 2 °C in 4 days, and then remained at 2 °C for another 8 days. Asterisks (*) indicate significant difference between low temperature as treatment and normal temperature as control at the same time points (*p* ≤ 0.05, *n* = 3, Student’s *t*-test).

Relative conductivity of litchi leaves increased with the chilling stress. From 4 d to 12 d of treatment, relative conductivity of the treated leaves was significantly higher than that of the controls, whereas the relative water content of the treated leaves was lower than that of the controls (Figure 3A,B). At day 12, the relative conductivity and relative water content of the treated leaves was 64.08% higher and 20.25% lower than that of the controls, respectively. In the treated leaves, activities of SOD, CAT, and POD showed increasing trends, while those of the control leaves were stable and remained at lower levels. The content of H_2_O_2_ in the treated leaves increased with the time of treatment and was significantly higher than that in the control leaves (Figure 3C–F).

### 2.3. Digital Transcriptomic Analysis

To identify genes in response to chilling stress in litchi, we collected leaves at 0 d, 4 d, 8 d, and 12 d time points of the low-temperature treatment and constructed 12 RNA-Seq libraries. As shown in Table 1, the percentage of Q30 of each sample was more than 93%.

### 2.4. Identification of Differentially Expressed Genes

**Figure 4:**
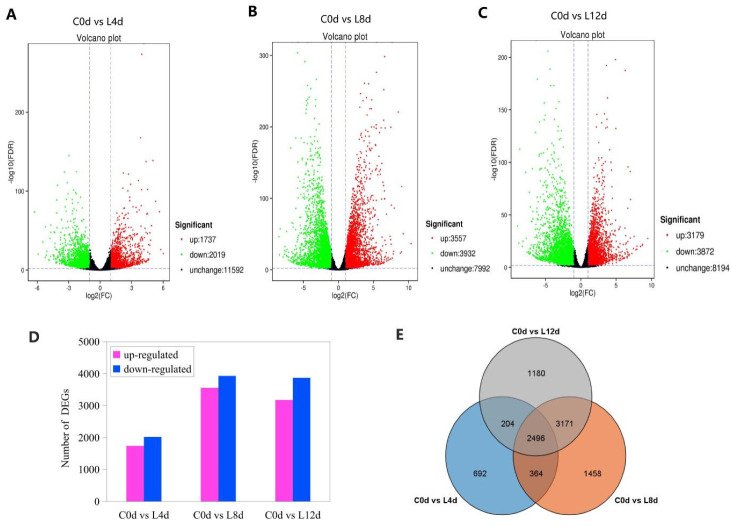
Differentially expressed genes (**A**–**D**) and Venn diagram of the DEGs (**E**) in response to chilling stress. ‘Huaizhi’ litchi trees were grown in a chamber with 22 °C/17 °C (day/night temperature, 12 h day and 12 h night) as the normal temperature. Temperatures dropped from the normal one to 2 °C for 4 d, and then were maintained at 2 °C for another 8 d. C0d, L4d, L8d, and L12d mean the leaves samples at 0 d, 4 d, 8 d, and 12 d of chilling stress.

On the basis of FDR ≤0.01 and fold-change ≥2, the DEGs of leaves between the chilling stress (L4d, L8d and L12d) and the control (C0d) were screened (Figure 4). Correlation analysis of the replicates of the four time points (0 d, 4 d, 8 d, and 12 d) showed high repeatability of the sequencing samples (Figure S1). On the whole, down-regulated DEGs were more than the up-regulated ones (Figure 4A–D). At last, we identified 3756, 7489, and 7051 DEGs from the comparison of the samples of 0 d to 4 d, 0 d to 8 d, and 0 d to 12 d, respectively, with an overlap of 2496 (Figure 4D). The 2496 DEGs were defined as chilling responsive genes (CRGs). Then, GO analysis was performed on the DEGs of each sample under chilling stress.

### 2.5. GO and KEGG Enrichment Analysis of the Differentially Expressed Genes

All DEGs were divided into three categories: biological process, cellular components, and molecular function. We found that the cellular process, the metabolic process, and the single-organism process were the most abundant in the category of biological processes (Table S1). In the category of cell elements, cell, cell parts, and membrane parts were the most abundant subcategories. Within the broad category of molecular functions, DEGs were enriched in protein binding, catalytic activity, and transporter activity (Figure S2).

**Figure 5:**
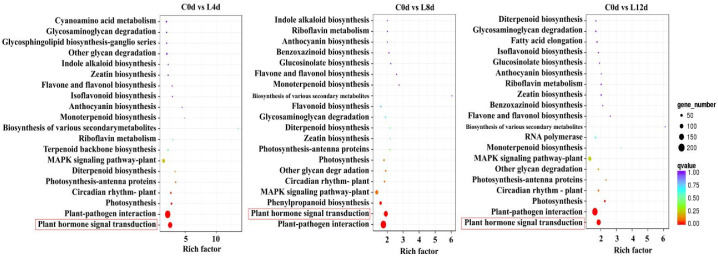
KEGG enrichment analysis of the DEGs. ‘Huaizhi’ litchi trees were grown in a chamber with 22 °C/17 °C (day/night temperature, 12 h day and 12 h night) as the normal temperature. Temperatures dropped from the normal one to 2 °C for 4 d, and then were maintained at 2 °C for another 8 d. C0d, L4d, L8d, and L12d mean the leaves samples at 0 d, 4 d, 8 d, and 12 d of chilling stress.

For KEGG pathway analysis, we found that the plant hormone signal transduction pathway was enriched in the DEGs between 0 d and 4 d, 0 d and 8 d, and 0 d and 12 d (Figure 5).

### 2.6. Identification of Plant Hormone Signal-Related Genes Involved in Chilling Stress

**Figure 6:**
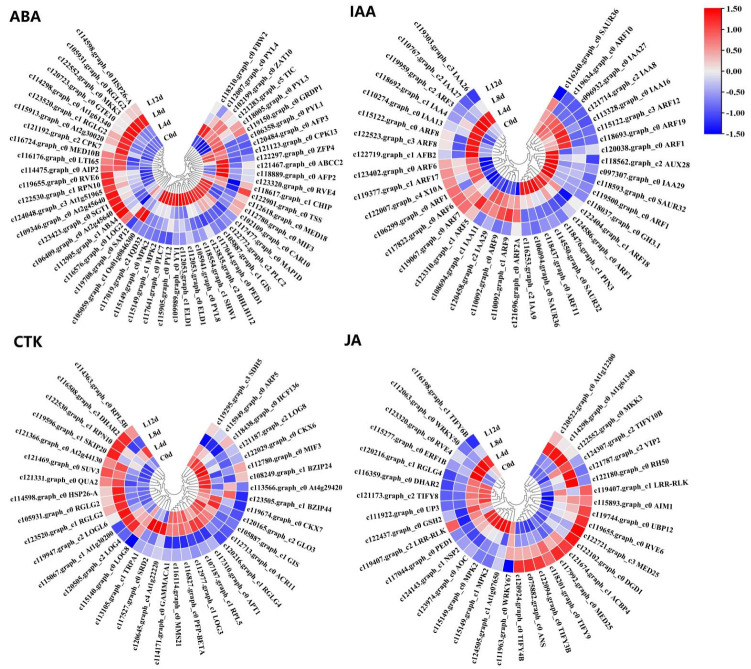
Heat map diagram showing the expression profiles of the ABA-, IAA-, CTK-, and JA-DEGs under low-temperature conditions. ‘Huaizhi’ litchi trees were grown in a chamber with 22 °C/17 °C (day/night temperature, 12 h day and 12 h night) as the normal temperature. Temperatures dropped from the normal one to 2 °C for 4 d, and then were maintained at 2 °C for another 8 d. C0d, L4d, L8d, and L12d mean the leaves samples at 0 d, 4 d, 8 d, and 12 d of treatment. Fragments per kilobase of transcript per million mapped reads (FPKM) values were normalized to Z-score.

**Figure 7:**
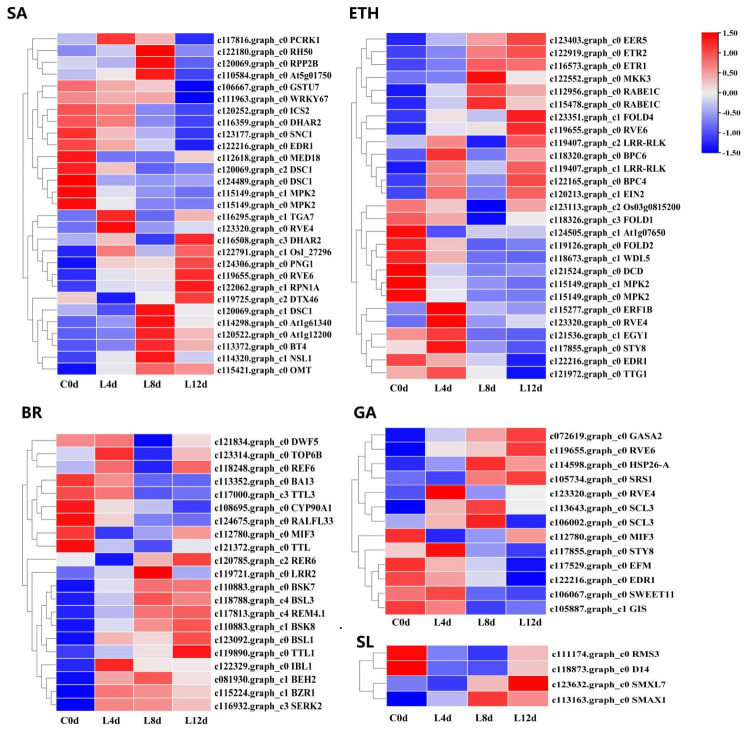
Heat map diagram showing the expression profiles of the SA-, ETH-, BR-, GA-, and SL-DEGs under low-temperature conditions. ‘Huaizhi’ litchi trees were grown in a chamber with 22 °C/17 °C (day/night temperature, 12 h day and 12 h night) as the normal temperature. Temperatures dropped from the normal one to 2 °C for 4 d, and then were maintained at 2 °C for another 8 d. C0d, L4d, L8d, and L12d mean the leaves samples at 0 d, 4 d, 8 d, and 12 d of treatment. Fragments per kilobase of transcript per million mapped reads (FPKM) values were normalized to Z-score.

From the 2496 CRGs, we screened those related to plant hormone signal transduction pathways (plant hormone signal transduction, ko04075), of which, 54 were related to abscisic acid signaling (ABA-DEGs), 40 were related to auxin signaling (IAA-DEGs), 37 were related to cytokinin signaling (CTK-DEGs), 27 were related to ethylene signaling (ETH-DEGs) (Figure 6), 21 were related to brassinolide signaling (BR-DEGs), 13 were associated with gibberellin signaling (GA-DEGs), 35 were associated with jasmonic acid signaling (JA-DEGs), 29 were associated with salicylic acid (SA-DEGs), and 4 were associated with strigolactone signaling (SL-DEGs) (Figure 7; Table S2).

### 2.7. Identification of Antioxidant Enzyme Encoding Genes Involved in Chilling Stress

**Figure 8:**
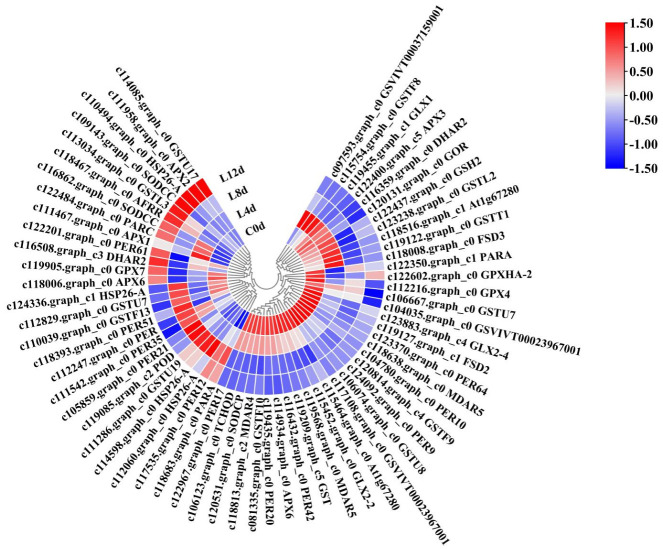
Heatmap of the antioxidant system-related DEGs in response to low-temperature in litchi. ‘Huaizhi’ litchi trees were grown in a chamber with 22 °C/17 °C (day/night temperature, 12 h day and 12 h night) as the normal temperature. Temperatures dropped from the normal one to 2 °C for 4 d, and then were maintained at 2 °C for another 8 d. C0d, L4d, L8d, and L12d mean leaves samples at 0 d, 4 d, 8 d, and 12 d of treatment. Fragments per kilobase of transcript per million mapped reads (FPKM) values were normalized to Z-score.

We focused on the KEGG pathways including ascorbate and aldarate metabolism, glutathione metabolism, peroxisome, phenylpropanoid biosynthesis, and pyruvate metabolism (Figure S3). These pathways contained genes related to the antioxidant system. As a result, we identified 63 DEGs (Figure 8) defined as antioxidant system-related CRGs (AO-CRGs) (Table S3).

### 2.8. Probable Pathway Involved in Hormonal Control in Response to Chilling Stress

From the CRGs, we also identified transcription factor encoding genes, including *LcICE1*, *LcCBFs*, and *LcbZIPs*. The expression patterns of these CRGs showed that they were all induced by the chilling stress (Figure S4). We also obtained the above nine types of hormone-related DEGs. We suspected that each type of hormone-related DEGs might be directly related to AO-CRGs to regulate litchi response to low temperature, or might be indirectly related to transcription factor-related CRGs (*LcICE1*, *LcCBFs*, and *LcbZIPs*) to affect AO-CRGs, and to regulate the responses of chilling stress in litchi. Hence, we constructed a model using PLS-SEM.

**Figure 9:**
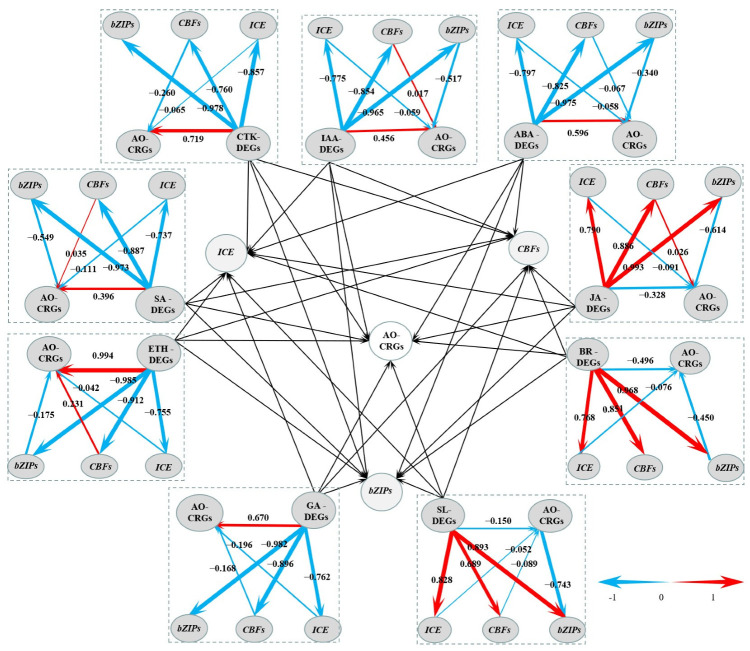
A graph of the Partial Least Squares Structural Equation Modeling (PLS-SEM). The center part of the graph is based on the other nine parts around it. Levels of the path coefficients are reflected by the width of the red or blue arrows which indicate positive and negative effects, respectively. Values on the arrow indicate the intensity of the factor loading for the pathway. Pathways from hormone-related DEGs to transcription factor-encoding DEGs are first orders, while those from transcription factor encoding DEGs to antioxidant-related CRGs are second orders.

Through PLS modeling data analysis, the influence of collinearity between variables could be effectively eliminated, and the relationship between observed variables and latent variables and between latent variables could be well tested. In the present study, most of the latent variables were significant, and the latent variables were adequately explained by the external model (Table S4). Meanwhile, the extracted mean variance (AVE, measure of convergent validity) was higher than 0.5 (Table S5), indicating that the model was reliable [18]. As shown in Figure 9, each model had two pathways, the direct pathway and the indirect pathway. In the indirect pathway, hormone-related DEGs had positive or negative effects on the three major transcription factors (TFs). In the ETH model, there were high factor loadings due to the high absolute value, such as −0.985, −0.912, and −0.755, higher than the second-order loads in the indirect path (−0.175, 0.231, and −0.042). This suggests that ABA-related DEGs were directly related to AO-CRGs. In addition, SL-DEGs, IAA, CTK, JA, BR, SA, ETH, and GA-DEGs were also directly related to AO-CRGs.

### 2.9. Confirmation of the RNA-Seq Data by qRT-PCR Analysis

**Figure 10:**
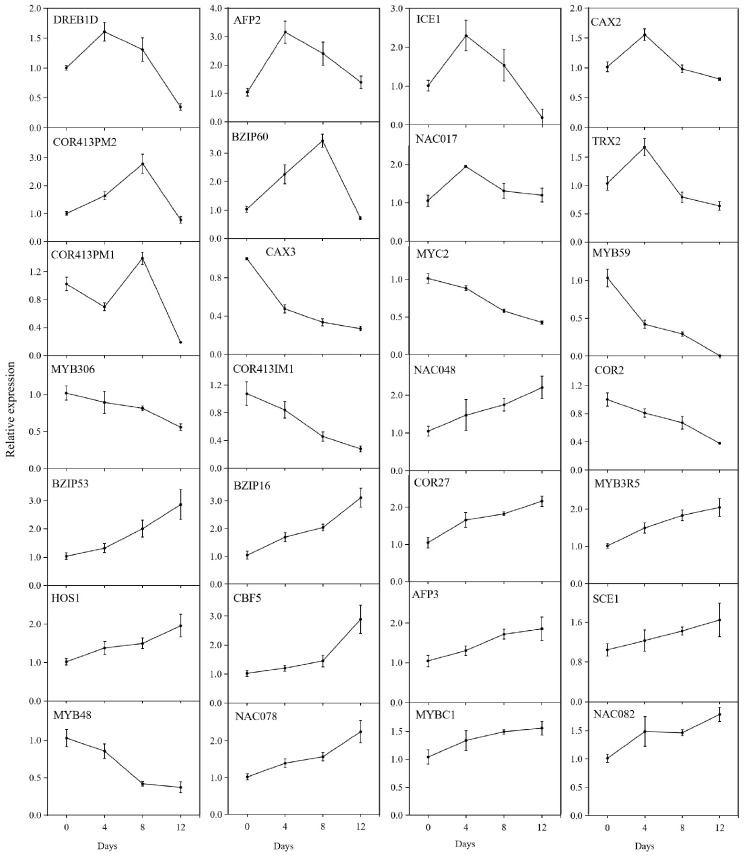
Expression of the 28 differentially expressed genes determined by quantitative real-time PCR. ‘Huaizhi’ litchi trees were grown in a chamber with 22 °C/17 °C (day/night temperature, 12 h day and 12 h night) as the normal temperature. Temperatures dropped from the normal one to 2 °C for 4 d, and then were maintained at 2 °C for another 8 d. C0d, L4d, L8d, and L12d mean the leaves samples at 0 d, 4 d, 8 d, and 12 d of treatment. Relative transcription was calculated by qRT-PCR using the 2^−ΔΔCT^ method with *actin* as a reference.

In order to confirm the RNA-Seq data, we randomly selected 28 DEGs for qRT-PCR verification. As shown in Figure 10, the relative expression levels of *LcDREB1D*, *LcAFP2*, *LcICE1*, *LcCAX2*, *LcNAC017*, and *LcTRX2* increased then decreased during the chilling treatment. The relative expression levels of *LcBZIP53*, *LcBZIP16, LcCOR27*, *LcMYB3R5*, *LcHOS1*, *LcCBF5*, *LcAFP3*, *LcSCE1*, *LcNAC048*, *LcNAC078*, *LcMYBC1*, and *LcNAC082* showed increasing trends.

**Figure 11:**
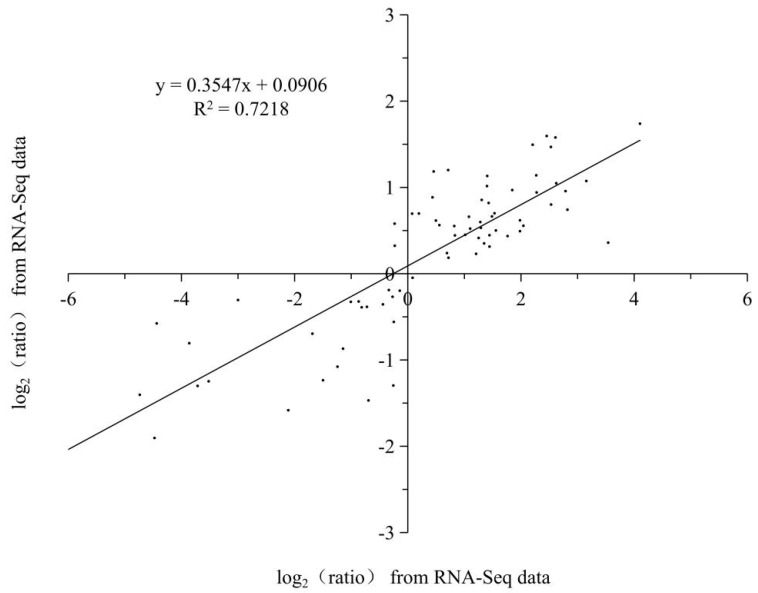
Coefficient analysis of fold change data between qRT-PCR and RNA-Seq. All the data are from the qRT-PCR analysis and RNA-Seq of the 0 d (C0d), 4 d (L4d), 8 d (L8d), and 12 d (L12d). Scatterplots were generated by the log2 expression ratios from RNA-seq (*x*-axis) and qRT-PCR (*y*-axis).

A linear regression analysis of the fold-change in the gene expression ratios between RNA-Seq and qRT-PCR showed a significantly positive correlation between the obtained data from qRT-PCR and RNA-Seq (Figure 11), confirming the reliability of our RNA-Seq data.

### 2.10. Model for the Litchi Response to Chilling

**Figure 12:**
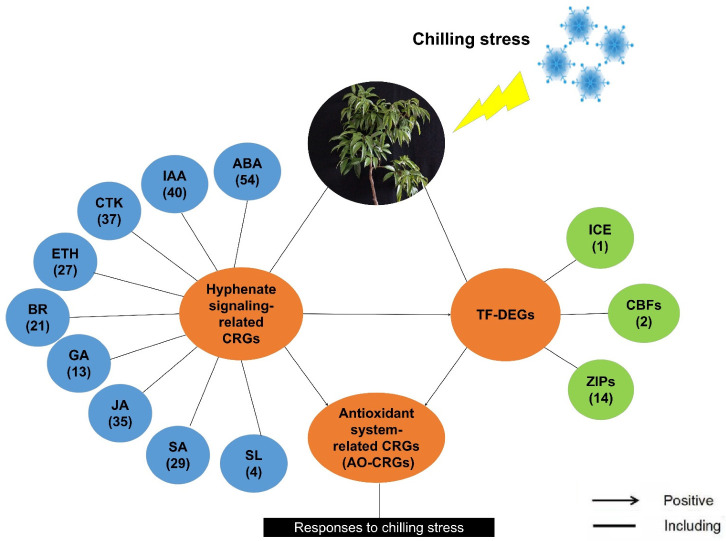
A proposed model for litchi in response to chilling according to the Partial Least Squares Structural Equation Modeling analyses. Details of the co-expression network of three identified cold-responsive genes and associated hormones are shown. The numbers in the brackets represent the number of the identified chilling responsive genes (CRGs) under the specific items.

We performed PLS-SEM analysis using RNA-Seq data and built a model to quantify their relationship. According to the PLS model, we proposed a model for litchi in response to chilling. As shown in Figure 12, when the litchi trees were subjected to chilling stress, the expression of plant hormone signal-related genes may change, directly affecting the expression pattern of the antioxidant system-related genes, or may regulate the expression of the *LcICE1*, *LcCBFs*, and *LcbZIPs*, and indirectly affecting the antioxidant system-related genes. As a result, *LcICE1*, *LcCBFs*, and *LcbZIPs* may affect the antioxidant system-related genes. The antioxidant system of the litchi trees may be activated, helping the trees to react and adapt to chilling stress.

## 3. Discussion

It has been shown that the stability of the cell membrane system indicated by the related conductivity may be affected under chilling stress [19,20]. The membrane system is the site of temperature sensing when plants are exposed to low temperatures [21]. The chlorophyll fluorescence parameter Fv/Fm reflects the maximum quantum efficiency of photosystem II (PSII) photochemistry and has been widely used for stress detection in plants [22]. In the present study, the Fv/Fm value showed a decreasing trend while relative conductivity showed an increasing trend, suggesting that litchi trees were affected by the chilling stress.

The antioxidant system is a key protection mechanism for plants under stressful conditions [23]. Activities of SOD, POD, and CAT, which belong to the antioxidant system, were determined. The results showed that they increased continuously with the time of the chilling treatment, indicating that the antioxidant system of litchi was activated under chilling conditions. However, we still detected an increasing accumulation of H_2_O_2_. It is well known that H_2_O_2_ can be removed by antioxidant enzymes [24]. It might be that the balance of the synthesis and scavenging of H_2_O_2_ was broken by chilling stress [25].

To study the transcriptomic changes in litchi under chilling stress, we sequenced the leaf samples at four time points of the low-temperature treatment and obtained 2496 CRGs. Interestingly, the KEGG enrichment analysis showed that the plant hormone signal transduction pathway was significantly enriched. Plant hormones are trace organic compounds that are induced by plant cells to receive environmental signals and act as signaling molecules [26]. At low concentrations, they can regulate the formation of flowers, stems, and leaves, control the development and ripening of fruits, and regulate the physiological responses of plants to stresses [27]. Our results suggest that plant hormone signal transduction might be an important strategy for litchi to adapt to chilling stress.

The plant hormone abscisic acid (ABA) is known as a signaling substance and is induced by abiotic stresses [28,29]. Abscisic acid receptor kinase 1 (CARK1) phosphorylates RCAR/PYR/PYLs and enhances the inhibition of PP2Cs, leading to the activation of abscisic acid signaling under stress [30]. Aux/IAA proteins are a large family of auxin co-receptors and transcriptional repressors that play central roles in auxin signaling [31]. GH3 encodes an IAA-amino acid synthase which can prevent IAA accumulation and regulate plant growth at low temperatures through an auxin-dependent pathway. In this study, we also identified ABA and IAA signaling transduction component encoding genes in litchi, such as *LcABA4*, *LcIAA9*, and *LcGH3.1*. They were differentially expressed under chilling conditions.

The cytokinin signaling pathway is based on a binary signaling system which is achieved through the continuous transmission of phosphate groups between major components [32]. The loss of function or the mutation of LONELY GUY (LOG), which encodes a cytokinin-activating enzyme, delays root growth in plants under extremely low-temperature conditions [33]. The fine-tuning of ethylene content in tissues affects plant growth and development [34]. Liu et al. [35] found that ERF9 could be induced by ethylene under low-temperature conditions [36]. The transmission of the brassinosteroid signal finally makes the transcription factor family BES1/BZR1 accumulate in a non-phosphorylated state, which activates downstream transcriptional regulation in the nucleus. It then regulates the growth and development of plants. In contrast, the AP2/ERF transcription factor TINY inhibits brassinolide-regulated growth [37]. From the CRGs, we identified four LOG encoding genes, *LcLOG3*, *LcLOG4*, *LcLOGL6*, and *LcLOG8*, in litchi. We also identified *LcERF1B*, and *LcBZR1D*. Expression patterns of these genes showed that they were chilling responsive. Other phytohormone signaling-related genes such the GA, SA, and SL signal-related CRGs in litchi were shown to be chilling-responsive, indicating that phytohormone signals play important roles in the response to chilling in litchi.

As a transmission signal, plant hormones can be directly transmitted to the nucleus to activate the expression of genes to cause corresponding physiological and biochemical changes in plants. Transcription factors can bind to specific DNA sequences to control the expression of particular genes. ICE1 [38,39], CBFs [35,40], and bZIPs [41,42,43,44] are central transcription factors downstream of plant hormone signaling transduction, and act as executors for the hormonal controlled plant growth and development. We hypothesize that they may play an important role in the response of chilling stress in litchi, and might act as mediators in phytohormone signaling and the antioxidant system to regulate the response of litchi to chilling stress. In our previous study, we used PLS-SEM for the first time to elucidate a pathway for high-temperature-induced floral abortion in litchi’s underlying gene expression [17]. In accordance with our previous study, this study further shows that PLS-SEM is valuable in revealing relationships between biological processes based on RNA-Seq data. Future studies should focus on the *ICE1*, *CBFs*, and *bZIPs* underlying the regulation of chilling responses of plant hormones in litchi.

## 4. Materials and Methods

### 4.1. Experimental Procedure

Seven-year-old ‘Huaizhi’ litchi trees were planted in 30 L pots containing loam, mushroom cinder, and coconut chaff (3:1:1 (*v*/*v*/*v*)). The trees were cultivated in the experimental orchard of South China Agricultural University, Guangzhou, China (lat. 23°90′40″ N, long. 113°210′18″ E). The trees with mature leaves in the terminal shoots were selected. The trees were transferred to a growth chamber at 22 °C/17 °C (day/night temperature, 12 h day and 12 h night) with a relative humidity of 75–85% and a light intensity of 120 µmol·m^−2^ s^−1^. The program for the chilling treatment is shown in Table 2, according to our previous studies [45]. The temperature was maintained at 2 °C from day 4 until the end of the experiment. The fourth and fifth mature compound leaves of the terminal shoots were collected every 2 days. Samples were frozen in liquid nitrogen immediately and stored at −80 °C.

### 4.2. Determination of Maximal Photochemical Efficiency

Leaves were dark-adapted for 20 min, and then the initial fluorescence yield (Fo), maximal fluorescence yield (Fm), and maximal photochemical efficiency (Fv/Fm) were measured by a chlorophyll fluorescence imaging system (Model IMAGING-PAM, WALZ company, Effeltrich, Germany).

### 4.3. Determination of Relative Conductivity and Relative Water Content

The relative conductivity analysis was preformed according to the method of Redmann et al. [46]. First, 0.1 g of fresh leaves was incubated in 20 mL of distilled water for 12 h, measured with a conductivity meter (Model DDS-307, Shanghai Leici Instrument Inc., Shanghai, China), heated in boiling water for 30 min, cooled to room temperature, and measured for conductivity again. The relative conductivity of the sample was calculated according to the ratio of the conductivity before and after boiling.

Fresh leaves (0.1 g) were kept in distilled water for 24 h and weighed. The leaves were then dried and weighed. Relative water content was calculated as described by Weatherley [47].

### 4.4. Determination of Activities of Superoxide Dismutase, Catalase, and Peroxidase

Next, 1.0 g leaves were taken and 50 mM (pH 7.8) phosphate buffer in 10 mL was added, then centrifuged at 10,000× *g* for 20 min at 4 °C. The supernatants were used for the determination of enzyme activity.

Superoxide dismutase (SOD) activity was measured according to the nitrogen blue tetrazolium (NBT) method [48]. The reaction solution containing 50 mM PBS, 130 mM methionine, 75 µM NBT, 50 µM EDTA-Na_2_, and crude enzyme solution was illuminated at 80 µmol·m^−2^ s^−1^ for 25 min. The absorbance was monitored at 560 nm wavelength by a spectrophotometer (Fluoroskan Ascen FL, Thermo, Waltham, MA, USA).

Catalase (CAT) activity was determined by UV absorption method [49,50]. The 3 mL reaction solution contained 15 mM H_2_O_2_, 50 mM phosphate buffer, and crude catalase extracts. Catalase activity was determined according to the decrease in the absorbance of H_2_O_2_ at 240 nm.

Peroxidase (POD) activity was measured using the guaiacol method described by Gillikin and Graham [51]. The 3 mL reaction solution contained 25 mM guaiacol, 10 mM hydrogen peroxide, 50 mM (pH 7.8) phosphate, and crude extracts. Changes in the absorbance of the reaction solution at a wavelength of 470 nm were determined by a spectrophotometer (Fluoroskan Ascen FL, Thermo).

### 4.5. Determination of Hydrogen Peroxide Content

The H_2_O_2_ content was determined using a commercial kit according to the manufacturer’s instructions (Jiancheng, Nanjing, China). The absorbance measurements were performed using a spectrophotometer (Fluoroskan Ascen FL, Thermo).

### 4.6. RNA Isolation, cDNA Library Construction, and Analysis

Total RNA was extracted using the RNAprep Pure Plant Kit (polysaccharide and polyphenolic-rich) (Tiangen Biotech, Beijing, China). First-strand cDNA was synthesized using a Reverse Transcriptase M-MLV (RNase H-) system (Takara, Dalian, China) from 1 μg extracted RNA. Sequencing libraries were generated using NEBNext^®^Ultra™ RNA Library Prep Kit for Illumina^®^ (NEB, Ipswich, MA, USA) following the manufacturers’ instructions. Briefly, Oligo-dT beads (Qiagen, Valencia, CA, USA) were used to enrich mRNA. After fragmentation, the fragmented mRNA was used as a template to synthesize single-stranded cDNA with hexamer random primers. Second-strand cDNA synthesis was subsequently performed using DNA Polymerase I and RNase H. In order to select cDNA fragments of preferentially 240 bp in length, the library fragments were purified with an AMPure XP system (Beckman Coulter, Beverly Hills, CA, USA). Then, PCR was performed with Phusion High-Fidelity DNA polymerase, Universal PCR primers, and Index (X) Primer. At last, PCR products were purified (AMPure XP system), and library quality was assessed on the Agilent Bioanalyzer 2100 system.

### 4.7. RNA-Seq Data Analysis

Adapters were cut from raw reads, and the low-quality sequences were removed. rRNA sequences were filtered against ribosomal RNA using Bowtie2 (version 2.1.0., Bowtie2. unpublished work. Available online: http://litchidb.genomics.cn, accessed on 12 June 2021). The read counts and fragments per kilobase of the transcript per million mapped reads (FPKM) of each gene were calculated using eXpress (v1.5.1, eXpress. Available online: https://pachterlab.github.io/eXpress/index.html, accessed on 12 June 2021). Q20, Q30, GC-content and sequence duplication level of the clean data were calculated. All the downstream analyses were based on clean data with high quality. The differential expression analysis of two groups was performed using the DESeq R package (1.10.1). Significant DEGs were restricted with false discovery rate (FDR) ≤0.01 and the absolute value of fold-change ≥2 [52]. Gene ontology (GO) enrichment analysis of the DEGs was implemented by the topGO R package-based Kolmogorov–Smirnov test. The enrichment of the GO functional classifications was plotted using the R Bioconductor package GOstats [53]. KEGG enrichment analysis was performed using a website of https://www.genome.jp/kegg/, accessed on 16 June 2021. The dataset is available in the NCBI Short Read Archive (SRA) under the accession number PRJNA845931.

### 4.8. Quantitative RT-PCR Analysis

Quantitative real-time polymerase chain reaction (qRT-PCR) primers F1/R1 (Table S6) were designed by Primer 6.0 (Premier Biosoft, Palo Alto, CA, USA) and synthesized by Sangon Co., Ltd. (Shanghai, China). The qRT-PCR was performed on a CFX96 real-time PCR machine (Bio-Rad, Hercules, CA, USA) with 2×RealStar Green Power Mixture (GenStar BioSolutions, Beijing, China). qPCR was performed at 95 °C for 10 min followed by 40 cycles of 95 °C for 15 s, 60 °C for 30 s, and 72 °C for 30 s in 96-well optical reaction plates. Each qRT-PCR analysis was performed in three biological and three technical replicates. The transcript quantification of the genes was performed in relation to Actin, and was calculated using the 2^−∆∆CT^ method [54].

### 4.9. Statistical Analysis

The IBM SPSS statistical software 23.0 was used to determine significant differences between the treatment and controls. The expression of genes was presented as a heat map diagram using the pheatmap package (http://www.r-project.org/, accessed on 24 June 2022). To explore the relationships between DEGs and the responses of litchi to low temperature, a hypothetical model according to Chen et al. [55] was specified and analyzed with PLS-SEM with the support of the SmartPLS 2.0 M3 software [56]. Standardized path coefficient values were generated with the PLS algorithm by the Path Weighting Scheme using a bootstrapping method to obtain the significance of path coefficients. The sign changes were individual changes. The samples during the calculation of the bootstrapping method were 5000, and the ABA-, IAA-, CTK-, JA-, SA-, ETH-, BR-, GA-, SL-related DEGs models were 54, 40, 37, 35, 29, 27, 21, 13, 4, respectively.

## 5. Conclusions

Twelve RNA-Seq libraries of the leaf samples were constructed at 0, 4, 8, and 12 d of chilling treatment. We screened 2496 CRGs. From this, 260 plant hormone signal transduction-related CRGs were identified. PLS-SEM analysis of the RNA-Seq data suggested a model for the litchi plants in response to chilling stress by altering the expression of plant hormone signal-related genes, the transcription factor encoding genes *LcICE1*, *LcCBFs*, and *LcbZIPs*, and the antioxidant system-related genes. Our study provides candidate genes for the future breeding of litchi cultivars with high chilling resistance, and elucidates possible pathways for litchi in response to chilling by PLS-SEM analysis using transcriptomic data.

## Figures and Tables

**Table 1:** Data quality and alignment analysis.

Sample	Base Sum	Read Number	Unique Match Reads	Mapping Rate (%)	Q20 (%)	Q30 (%)
C0d-1	10102123904	33801576	26241165	77.63	98.23	94.83
C0d-2	8453617060	28229953	22284855	78.94	98.22	94.69
C0d-3	8051853082	26899914	20872308	77.59	98.39	95.08
L4d-1	8695585806	29038679	22714867	78.22	98.27	94.81
L4d-2	7760353396	25907764	20542606	79.29	98.18	94.63
L4d-3	6884947538	22987219	17780526	77.35	98.39	95.14
L8d-1	8211342424	27401154	21548206	78.64	98.16	94.64
L8d-2	8704020992	29055786	23031477	79.27	98.24	94.88
L8d-3	7512882706	25103758	19619990	78.16	98.26	94.88
L12d-1	7258647484	24407112	18276685	74.88	97.83	94.12
L12d-2	6203789600	20737935	15970461	77.01	97.75	93.81
L12d-3	6625307298	22118812	17660747	79.84	97.98	94.39

The ‘Huaizhi’ litchi trees were grown in a chamber with 22 °C/17 °C (day/night temperature, 12 h day and 12 h night) as the normal temperature. Temperatures dropped from the normal one to 2 °C for 4 d, and then were maintained at 2 °C for another 8 d. The abbreviations in the first column of the table orderly mean 0 d (C0d), 4 d (L4d), 8 d (L8d), and 12 d (L12d) of low-temperature treatment. The Arabic numbers 1, 2, and 3 represent the samples of the 3 replications.

**Table 2:** The low-temperature program in the chamber.

Low-Temperature Time	Daily Variation of Temperature					
	1:00	8:00	12:00	15:00	19:00	22:00
0 d	22	22	24	20	20	17
1 d	17	17	20	16	16	12
2 d	12	12	15	11	11	7
3 d	7	7	9	5	5	2
4–12 d	2	2	2	2	2	2

‘Huaizhi’ litchi trees were grown in a chamber with 22 °C/17 °C (day/night temperature, 12 h day and 12 h night) as normal temperature. Temperatures dropped from the normal temperature to 2 °C for 4 d, and then were maintained at 2 °C for another 8 d.

## Data Availability

Data are available at NCBI SRA accession PRJNA845931.

## Supplementary Materials

The following supporting information can be downloaded at: www.mdpi.com/article/10.3390/ijms23158424/s1.
