# Supplementary material for: Identification of Chilling-Responsive Genes in Litchi chinensis by Transcriptomic Analysis Underlying Phytohormones and Antioxidant Systems
Source: Int J Mol Sci. 2022 Jul 29;23(15):8424. doi: 10.3390/ijms23158424 (PMC9369065; doi:10.3390/ijms23158424)
Supplement: Supplementary file 1 [file ijms-23-08424-s001.zip › Supplementary Figures S1 to S4.pdf]

Figure S1 Correlation analysis of biological replicates.

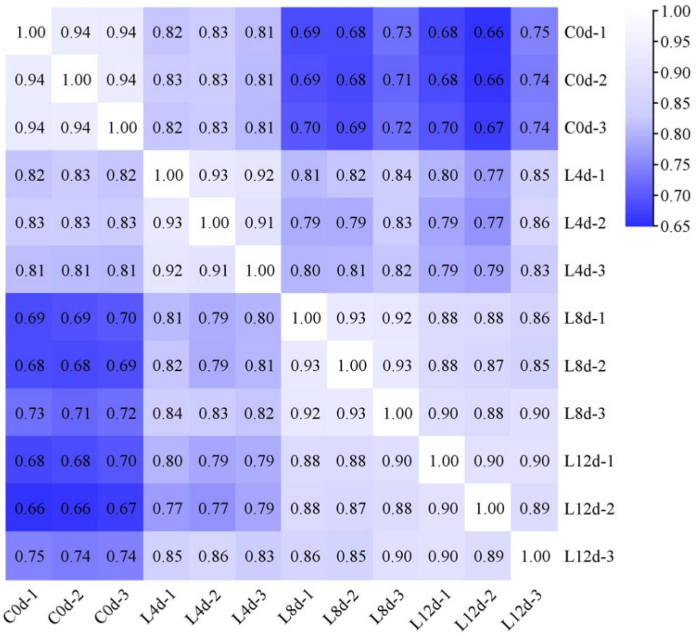

'Huaizhi' litchi trees were grown in a chamber at 22 °C/17 °C (day/night temperature, 12 h day and 12 h night) as normal temperature. Temperatures dropped from the normal one to 2 °C for 4 d, and then maintained at 2 °C for another 8 d. C 0 d, L 4 d, L 8 d, and L 12 d mean the leaves samples at 0 d, 4 d, 8 d, and 12 d of chilling stress.

Figure S2 The 20 top enriched GO terms of the DEGs in biological process (BP), cellular component (CC), and molecular function (MF) ontologies.

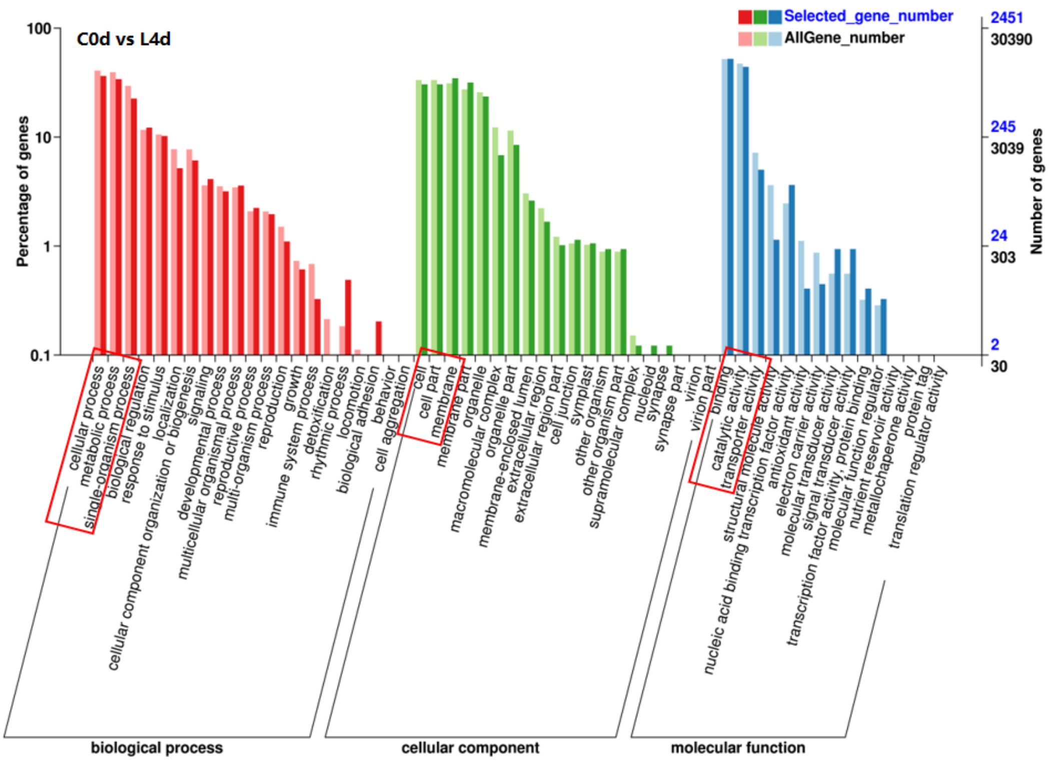

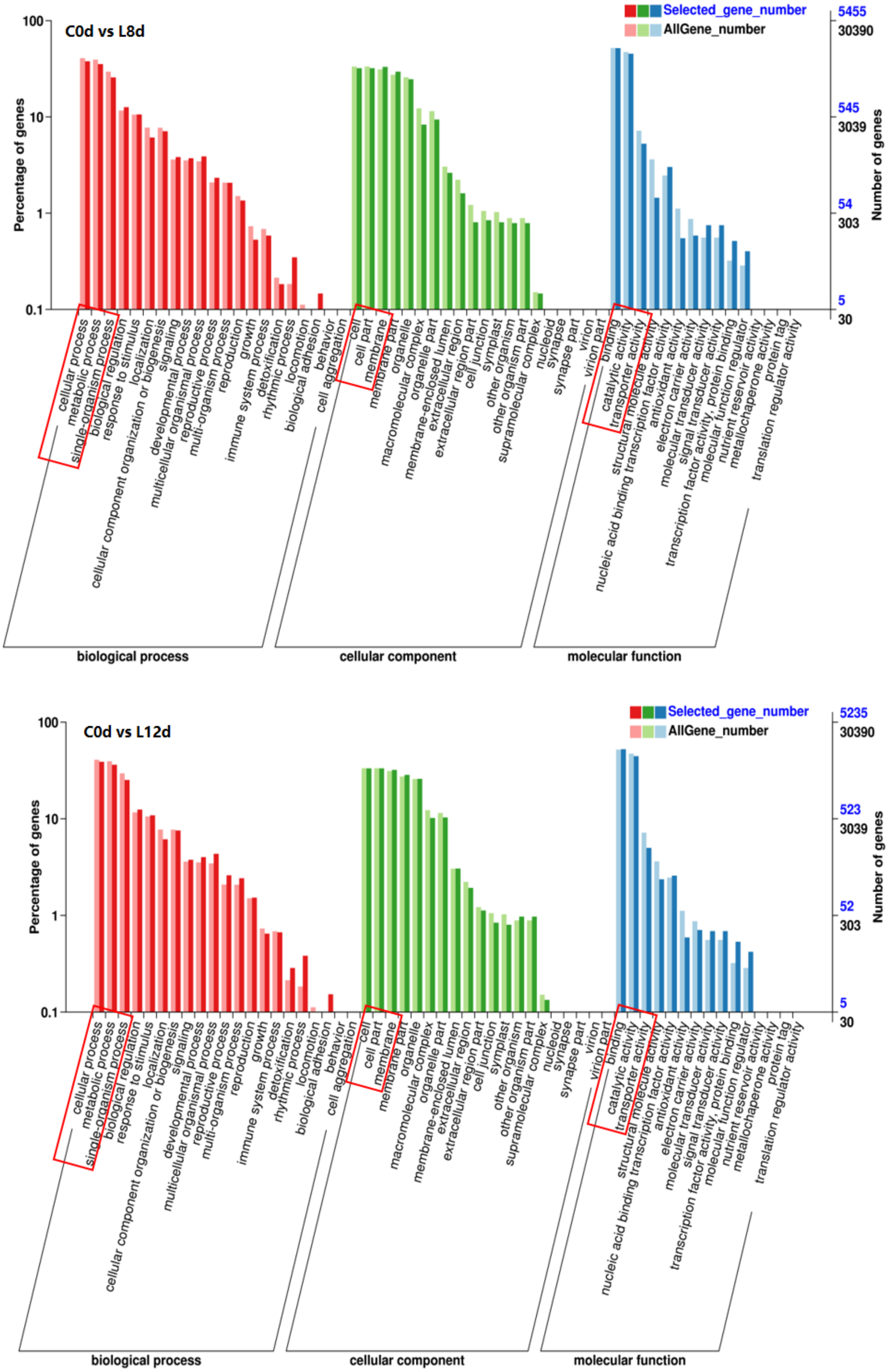

‘Huaizhi’ litchi trees were grown in a chamber at 22 ℃/17 ℃ (day/night temperature, 12 h day and 12 h night) as normal temperature. Temperatures dropped from the normal one to 2 ℃ for 4 d, and then maintained at 2 ℃ for another 8 d. C 0 d, L 4 d, L 8 d, and L 12 d mean the leaves samples at 0 d, 4 d, 8 d, and 12 d of chilling stress.

Figure S3 KEGG Pathway map：ascorbate and aldarate metabolism，glutathione metabolism, peroxisome, phenylpropanoid biosynthesis, pyruvate metabolism, plant hormone signal transduction.

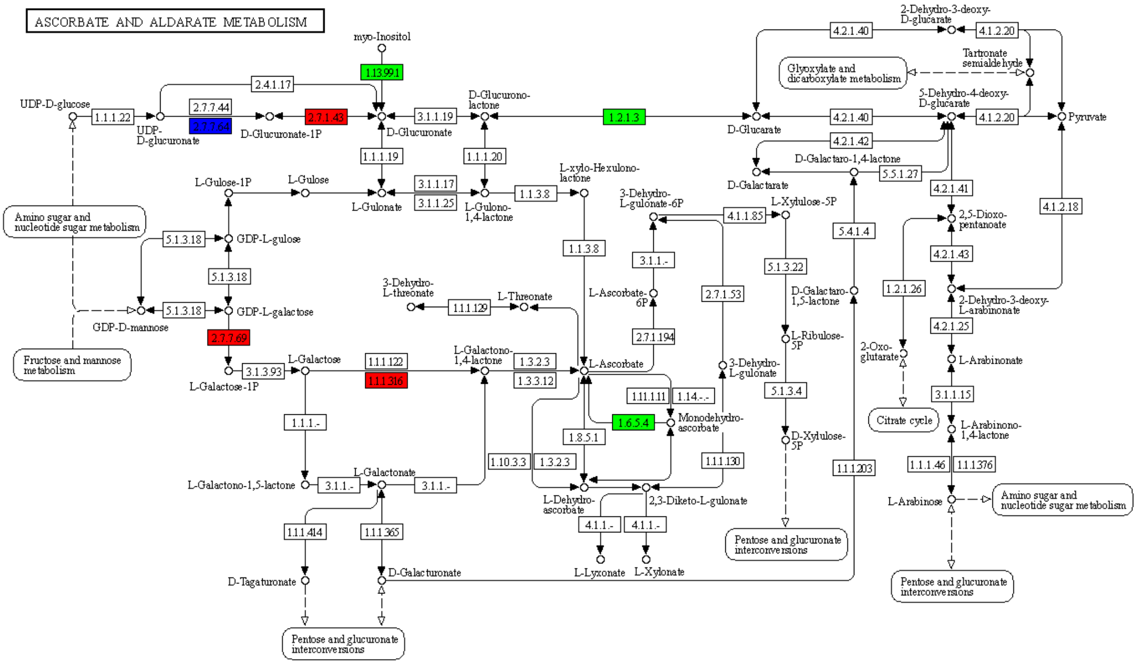

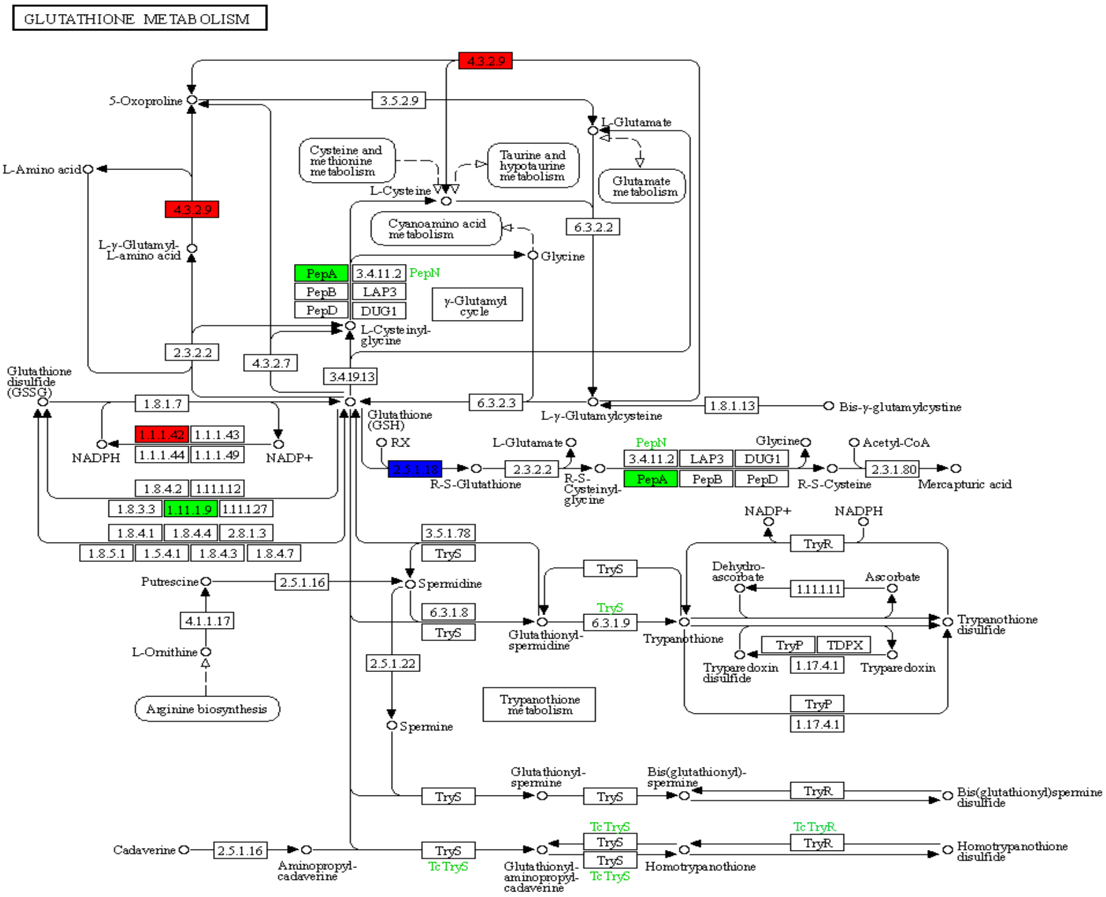

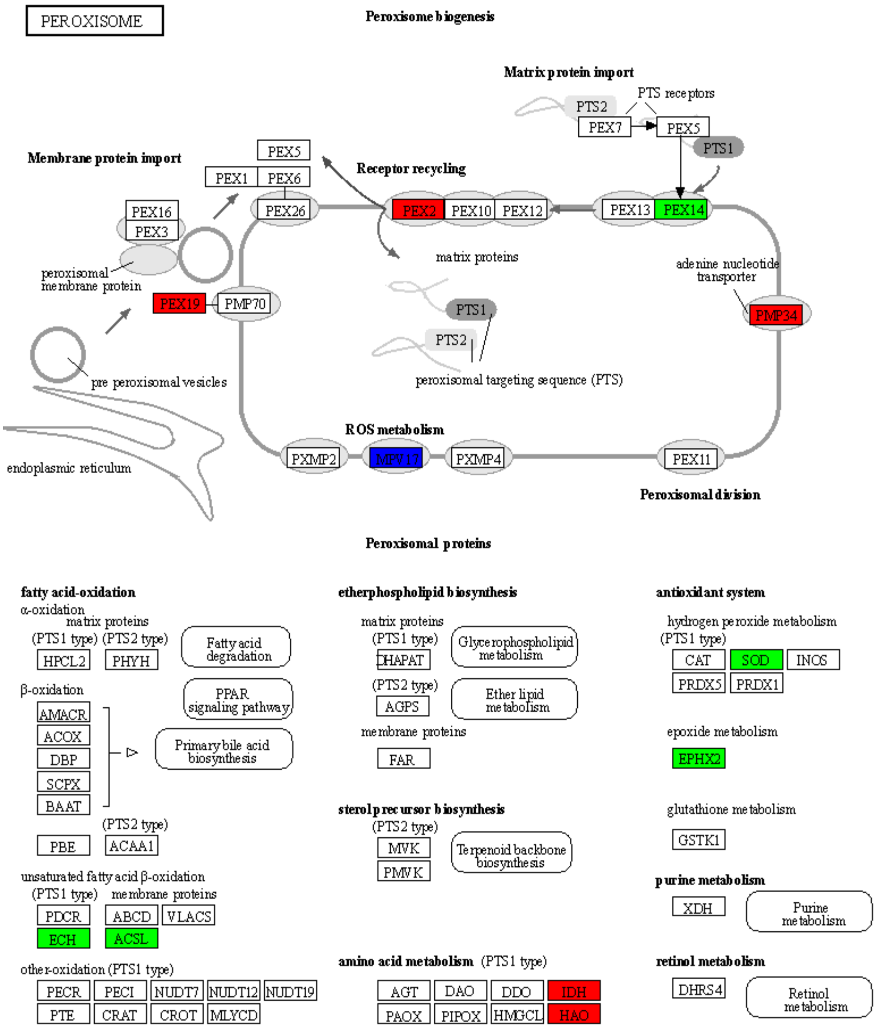
PEROXISOME
Peroxisome biogenesis
Matrix protein import
PTS receptors
Membrane protein import
Receptor recycling
matrix proteins
adenine nucleotide transporter
peroxisomal membrane protein
pre peroxisomal vesicles
peroxisomal targeting sequence (PTS)
endoplasmic reticulum
ROS metabolism
Peroxisomal division
Peroxisomal proteins
fatty acid-oxidation
α-oxidation
ether phospholipid biosynthesis
antioxidant system
hydrogen peroxide metabolism
β-oxidation
epoxide metabolism
sterol precursor biosynthesis
glutathione metabolism
purine metabolism
unsaturated fatty acid β-oxidation
other oxidation (PTS1 type)
amino acid metabolism (PTS1 type)
retinol metabolism

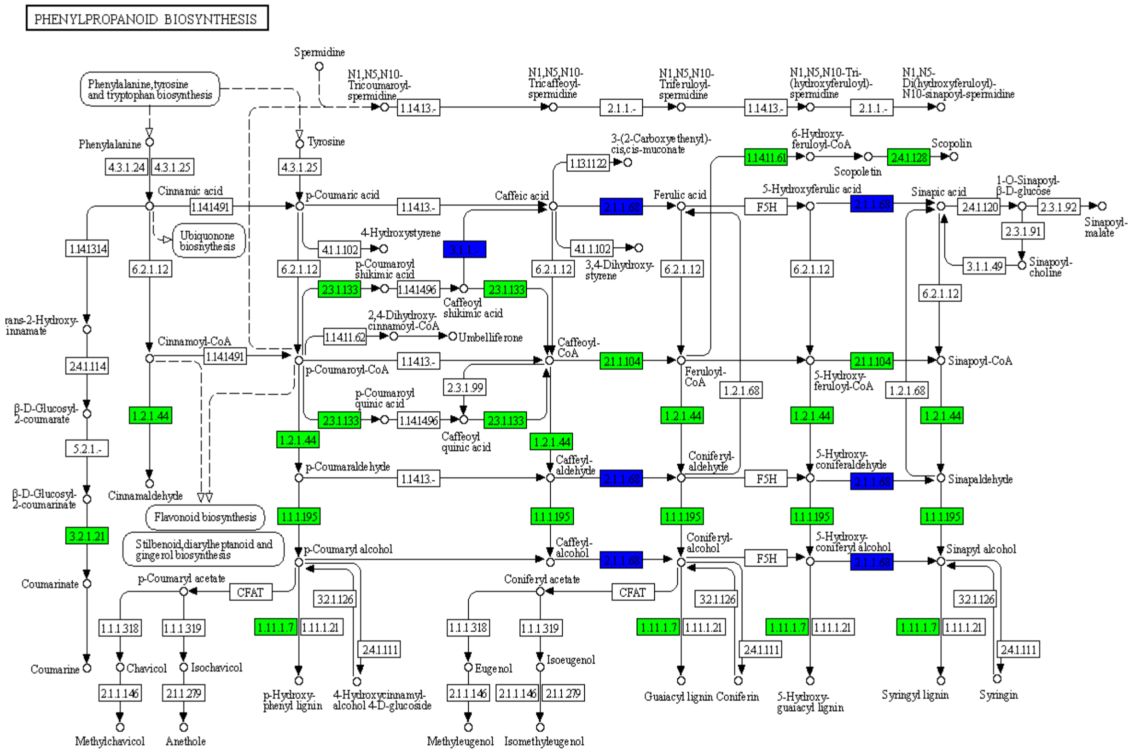
PHENYLPROPANOID BIOSYNTHESIS
Phenylalanine, tyrosine and tryptophan biosynthesis
Spermidine
Phenylalanine
Tyrosine
Cinnamic acid
p-Coumaric acid
Caffeic acid
Ferulic acid
5-Hydroxyferulic acid
Sinapic acid
Scopoletin
Umbelliferone
Cinnamoyl-CoA
p-Coumaroyl-CoA
Caffeoyl-CoA
Feruloyl-CoA
Sinapoyl-CoA
Cinnamaldehyde
p-Coumaraldehyde
Coniferyl-aldehyde
Sinapaldehyde
Flavonoid biosynthesis
p-Coumaryl alcohol
Coniferyl-alcohol
Sinapyl alcohol
Coumarinate
Coumarine
Chavicol
Isochavicol
Eugenol
Isoeugenol
Methylchavicol
Anethole
Methyleugenol
Isomethyleugenol
p-Hydroxy-phenyl lignin
Guaiacyl lignin
Coniferin
5-Hydroxy-guaiacyl lignin
Syringyl lignin
Syringin

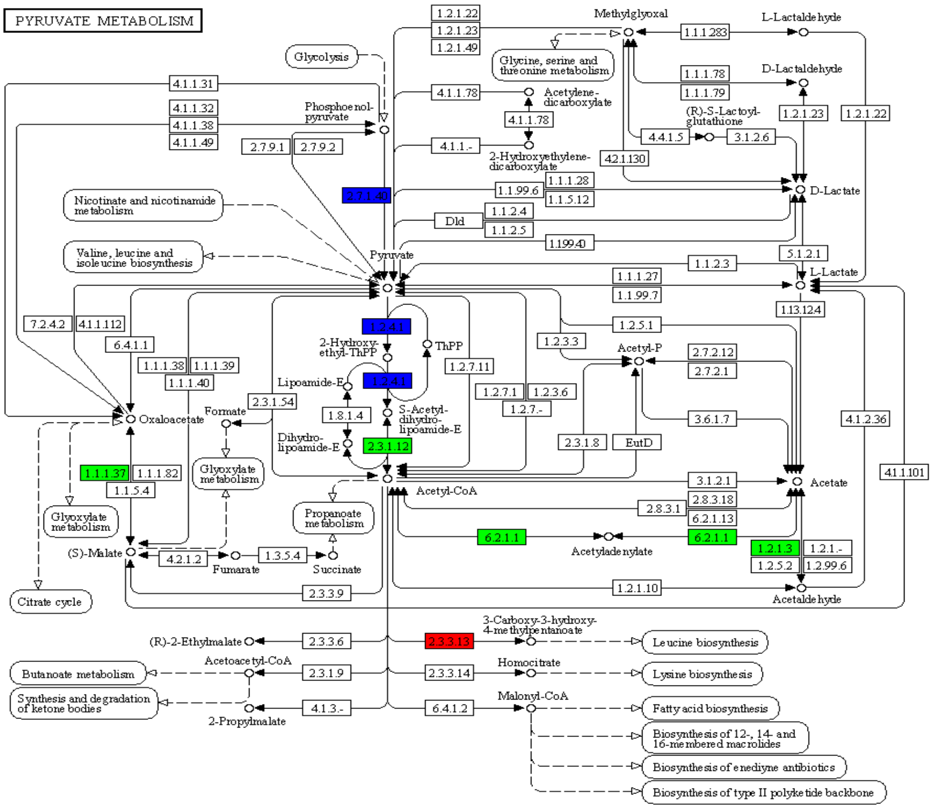

PYRUVATE METABOLISM
Glycolysis
Phosphoenol-pyruvate
Glycine, serine and threonine metabolism
Methylglyoxal
L-Lactaldehyde
D-Lactaldehyde
Acetylene-dicarboxylate
2-Hydroxyethylene-dicarboxylate
(R)-S-Lactoyl-glutathione
D-Lactate
L-Lactate
Nicotinate and nicotinamide metabolism
Valine, leucine and isoleucine biosynthesis
Pyruvate
2-Hydroxy-ethyl-ThPP
ThPP
Lipoamide-E
S-Acetyl-dihydro-lipoamide-E
Dihydro-lipoamide-E
Acetyl-CoA
Acetyl-P
Acetate
Acetyladenylate
Acetaldehyde
Oxaloacetate
Formate
Glyoxylate metabolism
Propanoate metabolism
(S)-Malate
Fumarate
Succinate
Citrate cycle
3-Carboxy-3-hydroxy-4-methylpentanoate
Leucine biosynthesis
(R)-2-Ethylmalate
Acetoacetyl-CoA
Butanoate metabolism
Synthesis and degradation of ketone bodies
2-Propylmalate
Homocitrate
Lysine biosynthesis
Malonyl-CoA
Fatty acid biosynthesis
Biosynthesis of 12-, 14- and 16-membered macrolides
Biosynthesis of enediyne antibiotics
Biosynthesis of type II polyketide backbone
1.2.1.22
1.2.1.23
1.2.1.49
1.1.1.283
1.1.1.78
1.1.1.79
4.1.1.31
4.1.1.32
4.1.1.38
4.1.1.49
2.7.9.1
2.7.9.2
4.1.1.78
4.1.1.-
4.4.1.5
3.1.2.6
4.2.1.130
2.7.1.40
1.1.99.6
1.1.1.28
1.1.5.12
Dld
1.1.2.4
1.1.2.5
1.1.99.40
1.1.2.3
5.1.2.1
1.1.1.27
1.1.99.7
1.13.12.4
1.2.4.1
1.2.5.1
1.2.3.3
2.7.2.12
2.7.2.1
1.2.7.11
1.2.7.1
1.2.3.6
1.2.7.-
3.6.1.7
4.1.2.36
2.3.1.8
EutD
2.3.1.12
1.8.1.4
2.3.1.54
7.2.4.2
4.1.1.112
6.4.1.1
1.1.1.38
1.1.1.39
1.1.1.40
1.1.1.37
1.1.1.82
1.1.5.4
4.2.1.2
1.3.5.4
2.3.3.9
3.1.2.1
2.8.3.18
2.8.3.1
6.2.1.13
6.2.1.1
1.2.1.3
1.2.1.-
1.2.5.2
1.2.99.6
1.2.1.10
4.1.1.101
2.3.3.6
2.3.3.13
2.3.1.9
2.3.3.14
4.1.3.-
6.4.1.2

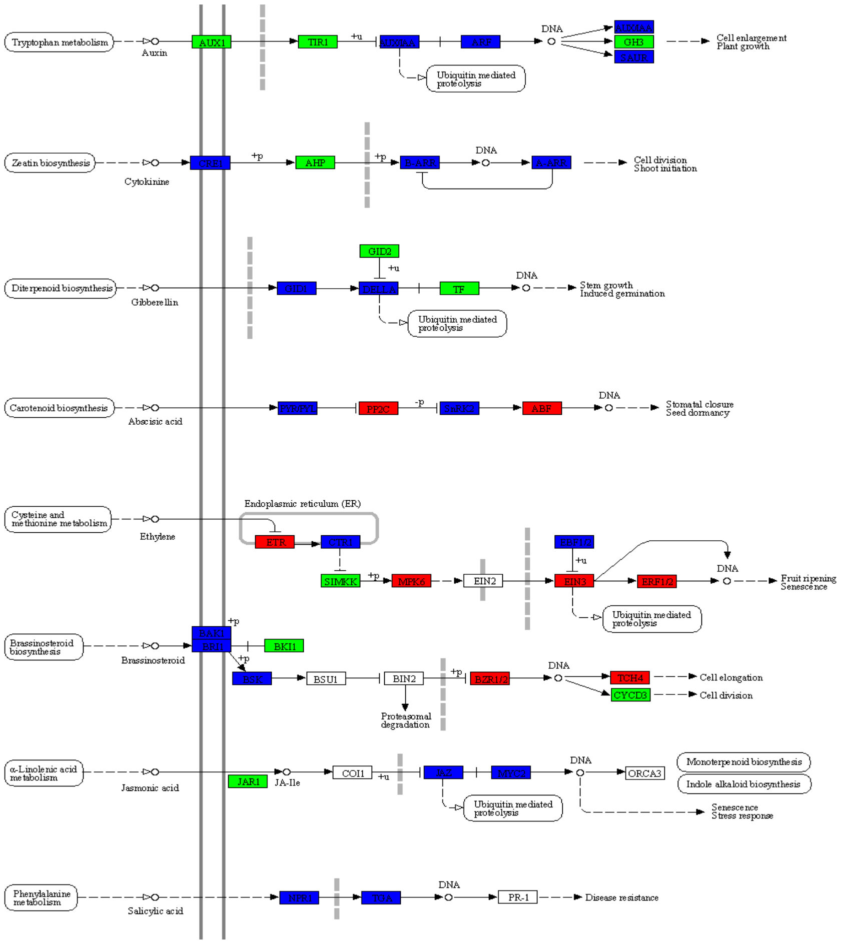

Tryptophan metabolism
Auxin
AUX1
TIR1
+u
ARF
DNA
GH3
Cell enlargement
Plant growth
Ubiquitin mediated proteolysis
Zeatin biosynthesis
Cytokinine
+p
AHP
DNA
Cell division
Shoot initiation
Diterpenoid biosynthesis
Gibberellin
GID2
+u
TF
DNA
Stem growth
Induced germination
Ubiquitin mediated proteolysis
Carotenoid biosynthesis
Abscisic acid
-p
DNA
Stomatal closure
Seed dormancy
Endoplasmic reticulum (ER)
Cysteine and methionine metabolism
Ethylene
SIMKK
+p
MPK6
EIN2
+u
EIN3
DNA
Fruit ripening
Senescence
Ubiquitin mediated proteolysis
Brassinosteroid biosynthesis
Brassinosteroid
+p
BKI1
+p
BSU1
BIN2
+p
DNA
Cell elongation
CYCD3
Cell division
Proteasomal degradation
α-Linolenic acid metabolism
Jasmonic acid
JAR1
JA-Ile
COI1
+u
DNA
ORCA3
Monoterpenoid biosynthesis
Indole alkaloid biosynthesis
Senescence
Stress response
Ubiquitin mediated proteolysis
Phenylalanine metabolism
Salicylic acid
DNA
PR-1
Disease resistance

Figure S4 Heat map diagram showing the expression profiles of DEGs identified from the four times during the low temperature stress.

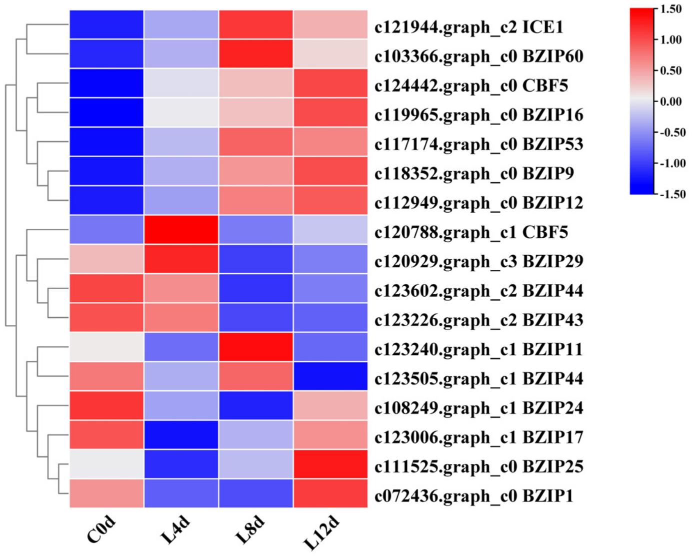

'Huaizhi' litchi trees were grown in a chamber at 22 ℃/17 ℃ (day/night temperature, 12 h day and 12 h night) as normal temperature. Temperatures dropped from the normal one to 2 ℃ for 4 d, and then maintained at 2 ℃ for another 8 d. C 0 d, L 4 d, L 8 d, and L 12 d mean the leaves samples at 0 d, 4 d, 8 d, and 12 d of treatment. Fragments per kilobase of transcript per million mapped reads (FPKM) values were normalized to Z-score.
